# Successful cognitive aging is associated with thicker anterior cingulate cortex and lower tau deposition compared to typical aging

**DOI:** 10.1002/alz.13438

**Published:** 2023-08-24

**Authors:** Stefania Pezzoli, Joseph Giorgio, Adam Martersteck, Lindsey Dobyns, Theresa M. Harrison, William J. Jagust

**Affiliations:** ^1^ Helen Wills Neuroscience Institute University of California Berkeley California USA; ^2^ Lawrence Berkeley National Laboratory Berkeley California USA; ^3^ University of Newcastle Newcastle NSW Australia

**Keywords:** Alzheimer's disease, amyloid, biomarkers, cortical thickness, exceptional cognitive performance, PET, successful aging, superaging, tau

## Abstract

**INTRODUCTION:**

There is no consensus on either the definition of successful cognitive aging (SA) or the underlying neural mechanisms.

**METHODS:**

We examined the agreement between new and existing definitions using: (1) a novel measure, the cognitive age gap (SA‐CAG, cognitive‐predicted age minus chronological age), (2) composite scores for episodic memory (SA‐EM), (3) non‐memory cognition (SA‐NM), and (4) the California Verbal Learning Test (SA‐CVLT).

**RESULTS:**

Fair to moderate strength of agreement was found between the four definitions. Most SA groups showed greater cortical thickness compared to typical aging (TA), especially in the anterior cingulate and midcingulate cortices and medial temporal lobes. Greater hippocampal volume was found in all SA groups except SA‐NM. Lower entorhinal ^18^F‐Flortaucipir (FTP) uptake was found in all SA groups.

**DISCUSSION:**

These findings suggest that a feature of SA, regardless of its exact definition, is resistance to tau pathology and preserved cortical integrity, especially in the anterior cingulate and midcingulate cortices.

**Highlights:**

- Different approaches have been used to define successful cognitive aging (SA).
- Regardless of definition, different SA groups have similar brain features.
- SA individuals have greater anterior cingulate thickness and hippocampal volume.
- Lower entorhinal tau deposition, but not amyloid beta is related to SA.
- A combination of cortical integrity and resistance to tau may be features of SA.

## 1 BACKGROUND

Successful cognitive aging (SA) can be conceptualized as one extreme of a continuum with patients with dementia at the other extreme and typical aging (TA) falling somewhere in the middle. Although most of the research in cognitive aging focuses on pathological and age‐related cognitive decline, studying SA is also crucial to uncover protective mechanisms.

Different approaches have been applied to the study of SA, focusing primarily on memory performance.^1^, ^2^, ^3^ Studies have described SA using different terms such as SuperAgers,^4^, ^5^, ^6^, ^7^ optimal memory performers,^8^ supernormals,^9^, ^10^, ^11^, ^12^, ^13^ superior memory performers,^14^ or high‐performing older adults,^15^, ^16^ and have used different criteria, including a minimum age and cognitive measures. Some SA definitions require memory performance comparable to published normative data of young or middle‐aged individuals.^4^, ^14^, ^17^, ^18^, ^19^ For example, one definition of SA as SuperAgers required individuals 80 years of age or older to show performance on the delayed verbal recall score of the Rey Auditory Verbal Learning Test at or above the average performance of middle‐aged adults 50 to 65 years of age.^4^, ^5^, ^6^, ^20^ Other studies used approaches similar to those with different age criteria of 70^14^, ^17^, ^21^ or 60^18^, ^19^, ^22^, ^23^, ^24^ years of age compared with younger adults 18 to 32^14^, ^22^, ^23^, ^24^ or 30 to 44 years of age.^17^, ^18^, ^19^ Another approach defines SA as older adults with higher cognitive performance (e.g., composite memory scores above the 80th or 90th percentiles) compared to individuals within the same age range.^8^, ^9^ Despite the broad methodological and conceptual differences in previous work examining SA, a comparison between SA definitions and related brain features has never been formally conducted.

Existing evidence on the neural substrates of SA is limited but suggests that SA is characterized by greater hippocampal volume and greater thickness in several regions of frontal cortex.^4^, ^8^, ^14^, ^22^ Less clear is the relationship with Alzheimer's disease (AD)–related pathology. Previous findings indicate that SA may be resilient to the negative effects of amyloid beta (Aβ), although the role of Aβ may be more detectable by longitudinal cognitive measures.^8^, ^14^ Neuropathological evidence suggests lower tau neurofibrillary tangles (NFTs) in SuperAgers compared with TA, but no differences in amyloid plaque density.^6^, ^25^ Despite these findings, evidence about the presence of AD pathology in SA is very limited.

A growing number of studies have used machine learning models to estimate brain age from structural neuroimaging data.^26^ The same method can be used to obtain age estimates based on individuals’ cognitive functioning, whereby a normative model is built using cognitive data to predict chronological age. Measuring cognitive‐predicted age is a novel approach that has been introduced only recently.^27^ This method has the potential to capture patterns of holistic cognitive aging that are not easily detectable by standard neuropsychological measures considered separately. SA can be defined as individuals deviating from normative cognitive trajectories independent of age, rather than being indirectly inferred using thresholds on single or composite measures of cognitive performance.

The present study aimed at using novel and existing approaches to define SA to examine underlying concepts of resistance to the emergence of AD pathology (i.e., SA showing lower levels of pathology than would be predicted by group‐level data) and resilience to its negative effects on cognition (i.e., SA showing comparable levels of pathology).^28^ To ensure that our findings were not driven by specific SA criteria, we considered different SA definitions. First, we developed a new measure to define SA using an age‐prediction model to calculate a cognitive age gap (CAG). Then we examined and compared SA definitions based on CAG, and more traditional measures of episodic memory (EM) and non‐memory cognition (NM) composites, and the California Verbal Learning Test (CVLT). By comparing different definitions, we were able to gain insights into whether the neurobiological characteristics linked to SA were influenced by the specific definitions used or if they were common across various SA groups. Finally, we explored differences between SA and TA in cortical thickness, hippocampal volume, and positron emission tomography (PET) measured amyloid and tau pathology.

**RESEARCH IN CONTEXT**

1. **Systematic review**: A systematic literature search was carried out using PubMed to identify articles investigating the brain features related to successful cognitive aging (SA). Despite the different approaches that have been used to define SA, a comparison between SA definitions and related brain features has never been formally conducted.
2. **Interpretation**: The results of this work indicate that different SA definitions identify only partially overlapping groups of older adults. Despite this, common brain features were found across definitions. These findings suggest that a feature of SA is resistance to tau pathology and preserved cortical integrity, especially in the anterior cingulate and midcingulate cortices.
3. **Future directions**: Longitudinal observations are needed to fully comprehend the many phenomena associated with SA. Moreover, it will be important in the future to investigate the relationship between both genetics and modifiable lifestyle factors related to SA and brain pathology.

## 2 METHODS

### 2.1 Sample

This study involved 531 community‐dwelling, cognitively unimpaired elderly participants from the Berkeley Aging Cohort Study (BACS) an ongoing longitudinal study of normal cognitive aging. Eligibility requirements included a baseline Mini‐Mental State Examination (MMSE) score ≥25, normal daily functioning, no history of neurological disease or major medical illness affecting cognition, and no history of substance abuse or depression. Participants remained cognitively normal throughout the study. The BACS cohort was divided into two groups of participants 55 years of age or older with a full battery of neuropsychological tests available: (1) BACS training cohort (*n* = 293) and (2) BACS test cohort (*n* = 238). Both cohorts contained similar participants and identical cognitive evaluations, but only those in the test cohort had neuroimaging data. The lack of neuroimaging scans in the training data set occurred because some participants did not meet criteria for imaging, and some declined to undergo neuroimaging. Individuals without imaging were also less likely to be recruited to longitudinal cognitive follow‐up. Additional requirements for the test cohort were T1 structural magnetic resonance imaging (MRI) and ^11^C‐Pittsburgh compound B (PiB) PET. A subgroup additionally had ^18^F‐Flortaucipir (FTP) PET scans. Other demographic and clinical features available included the following: years of education, apolipoprotein E (*APOE*) genotyping, history of hypertension, body mass index (BMI), self‐reported family history of dementia, and scores on the Geriatric Depression Scale (GDS). Details on the selection strategy are summarized in Figure S1.

The institutional review boards at the University of California, Berkeley, and the Lawrence Berkeley National Laboratory (LBNL) reviewed and approved the study. All participants provided written, informed consent for their participation in this study.

### 2.2 Neuropsychological assessment

The BACS protocol comprises a comprehensive neuropsychological battery assessing a variety of cognitive domains, including verbal and visual memory, working memory, processing speed, executive functioning, and attention. In this study the following tests were used: CVLT, Logical Memory, Visual Reproduction, Trail Making Test (TMT) A and B, Stroop test, digit symbol task, phonemic verbal fluency F‐A‐S test, Animal Naming, Vegetable Naming, Digit Span Forward and Digit Span Backward, and Boston Naming Test. Only participants who had been assessed with a full neuropsychological battery were included. Repeated neuropsychological assessment was available for 31% of the participants in the BACS training cohort (92 participants of 293 had two to nine completed neuropsychological sessions) and 86% in the BACS test cohort (205 participants of 238 had 2 to 12 completed sessions). In the training cohort, the mean ± standard deviation (SD) number of sessions was 1.56 ± 1.07 and mean follow‐up years was 0.97 ± 1.86; the mean number of sessions in the test cohort was 4.79 ± 3.00 and mean follow‐up years was 5.29 ± 3.98. Confirmatory factor analyses (CFAs) were used to calculate EM and NM cognition composite scores, as described in detail by Dobyns et al.^29^ The EM composite was quantified using the following tests: CVLT Short Delay Free Recall (SDFR), CVLT Long Delay Free Recall (LDFR), Visual Reproduction I, Visual Reproduction II, Logical Memory Total Score, and Verbal Paired Associates. The NM composite comprised the following: Stroop in 60 seconds, Digit Symbol, TMT‐A, TMT‐A subtracted from TMT‐B (Trails B–A), Digit Span Backward, Animal Naming, and Vegetable Naming.

### 2.3 Cognitive age model

To estimate cognitive‐predicted age, we used Partial Least Square regression (PLSr). PLSr is a multivariate method that reduces a set of predictor variables into latent variables (linear combinations of the original variables) that have maximum covariance with the response variables.^30^, ^31^ PLSr is well suited to prediction tasks where predictor variables are highly correlated,^32^ such as cognitive assessments, and allows for easy interpretation of the loadings within each dimension, representing key features with respect to the prediction task. PLSr was run setting the number of components to 5 using the plsregress.m function from the MATLAB statistics and machine learning toolbox. We trained the cognitive age (CA) model using an independent data set (BACS training cohort; *n* = 293; 55 to 96 years of age at baseline) than the data set used to associate with imaging variables (BACS test cohort; *n* = 238; 56 to 97 years of age at baseline). The model was trained on all neuropsychological sessions available for each participant in the training cohort, and the learned parameters were then used to predict age in the test cohort. The total number of sessions was 457 and 1141 in the training and test cohorts, respectively. The chronological age at each visit was used as the response variable and the following tests/subtests were used as predictor variables: CVLT Trials 1‐5 Free Recall total, CVLT SDFR, CVLT Short‐Delay Cued Recall (SDCR), CVLT LDFR, CVLT Long‐Delay Cued Recall (LDCR), TMT‐A and B, Stroop in 60 seconds, F‐A‐S test, Animal Naming, Vegetable Naming, Digit Symbol, Logical Memory total recall, Visual Reproduction I, II, and recognition total, Digit Span Forward and Backward, and Boston Naming Test (total number of tests/subtests: *n* = 19). Each test was variance normalized using the mean and SD of the training cohort prior to being modeled. To account for potential practice effects, session number for each neuropsychological session was also included as predictor variable (total number of predictors: *n* = 20). To assess prediction accuracy of chronological age by the model predicted age, we calculated the correlation between age and cognitive‐predicted age (Pearson correlation coefficient r), the total variance explained (*R* ^2^), and mean absolute error (MAE). We next applied a statistical bias correction to each individual's predicted age to account for a frequently observed bias in age prediction found at the tails of the distribution, resulting in an overestimated age for younger adults and underestimated age for older adults.^33^, ^34^, ^35^ In particular, we used a correction proposed by Beheshti et al.^36^ applied previously to brain age prediction models. First, we fitted the relationship between CAG (CAG = cognitive‐predicted age – chronological age) and chronological age using the following: Offset = α Ω + β, where Ω is chronological age, and α and β are, respectively, the slope and intercept of a linear regression model of CAG against chronological age.^33^, ^36^ This offset was then subtracted from each individual estimated cognitive‐predicted age to obtain bias‐corrected predicted age values.^33^, ^36^ This correction has been shown to provide age‐bias correction that is equivalent to that of other methods used to estimate brain age.^33^ Prediction accuracy metrics were reported prior age‐bias correction to provide a more accurate representation of the age‐prediction model performance.^34^ Age‐bias–corrected CAG scores were calculated by subtracting chronological age from the age‐bias–corrected cognitive age values. Finally, we assessed the weight stability of each predictor by generating 1000 bootstrapped samples from the training data set drawing with replacement. For each component, we normalized the mean weight across bootstrapped samples by dividing it by the corresponding SD (akin to a z statistic).

### 2.4 SA definitions

The magnitude of cognitive decline is heavily influenced by advancing age and, therefore, age is an important criteria to include in the definition of SA.^37^ For this reason, in the present study, only participants 70 years of age or older from the BACS test cohort (*n* = 184) were included to explore differences between SA and TA, in line with previous studies.^9^, ^14^, ^17^, ^21^ Cross‐sectional cognitive data closest to each participant's PiB scan were used to identify cutoffs to classify participants as either SA or TA. For 93% of participants, the time between the PiB scan and the cognitive session was ≤6 months; the time interval was less than a year for all subjects. Tau PET‐related analyses involved a subsample of participants (*n* = 114). The cognitive session closest to the FTP scan was used for tau PET‐related analyses. For 91% of participants, the time between the FTP scan and the cognitive assessment was ≤6 months; for only two participants, the time interval was slightly over a year (1.03 and 1.07 years). For this reason, all analyses were repeated including the time interval between scan date and cognitive session date as covariate of no interest in addition to age, sex, and years of education.

Individuals were identified as SA using four different definitions. The first definition was based on CAG scores. Consistent with the MRI brain age literature, negative CAG scores reflect younger cognitive age than chronological age. Therefore, participants with CAG scores within the lowest 20th percentile were defined as SA‐CAG. The cutoff was placed at the 20th percentile to be consistent with previous approaches defining SA as participants with cognitive performance in the top 20%.^8^ The second definition was similar to SA definitions used previously based on the performance on memory composite scores in comparison with average performance of individuals with the same age.^8^, ^9^ SA was defined as individuals with performance in the top 20% at the age‐adjusted CFA episodic memory composite described above. The cutoff score was set at the 80th percentile of age‐regressed residuals. Similarly, a third definition was based on performance on age‐adjusted CFA non‐memory cognition composite scores, placing a cutoff score for SA at the 80th percentile of age‐regressed residuals. In the first, second, and third definitions, the larger cohort of participants 70 years of age or older with PiB data available (*n* = 184) was used to identify percentile‐based cutoff values to classify participants into SA/TA groups in all analyses. The fourth definition was based on the comparison with average normative data for younger populations at the CVLT LDFR, as previous definitions.^14^, ^17^, ^22^ In particular, we defined SA as participants with a performance comparable to individuals 18 to 32 years of age (score of 14 or above; max score = 16).^14^, ^22^ All individuals in the cohort not meeting any of the four SA criteria were defined as typical agers. In all analyses comparing SA and TA, therefore, each SA group was compared with the same group of typical agers (*n* = 110).

### 2.5 MRI acquisition and processing

Each participant within the test cohort underwent a high‐resolution T1‐weighted magnetization‐prepared rapid gradient echo (MPRAGE) scan acquired on a 1.5 T Siemens Magnetom Avanto scanner at LBNL. The following acquisition parameters were applied: repetition time (TR) = 2110 ms, echo time (TE) = 3.58 ms, flip angle = 15°, 1 mm slice thickness, and 1 × 1 mm^2^ in‐plane resolution. For each participant, we selected the MRI scan closest to the PiB scan. MRI and PiB scans were acquired within 6 months for all subjects. The time between the MRI scan and the cognitive session used to define SA/TA groups was ≤6 months for 91% of participants and was less than a year for all participants. T1‐weighted MPRAGE scans were processed using FreeSurfer version 5.3 (http://freesurfer.net/).^38^, ^39^, ^40^ Briefly, FreeSurfer reconstructs three‐dimensional (3D) pial and white matter surfaces based on the relative intensity differences at the boundaries of each tissue class. Cortical thickness is calculated across ≈150,000 vertices per hemisphere as the average distance of the vectors perpendicular to the triangular faces of the white matter and pial surfaces.^38^ The FreeSurfer volumetric segmentation was used to calculate hippocampal volumes and total intracranial volume (TIV).^41^

### 2.6 PET acquisition and processing

Participants within the test cohort underwent Aβ‐PET imaging with PiB and tau‐PET imaging with FTP conducted on a BIOGRAPH PET/CT scanner using protocols as detailed in previous publications.^42^, ^43^, ^44^ PiB and FTP were synthesized at the LBNL Biomedical Isotope Facility.

For PiB‐PET images, 90 minutes of dynamic emission data frames was acquired after an injection of 15 mCi of PiB tracer. A computerized tomography (CT) scan was obtained pre‐injection and used for attenuation correction. PiB‐PET images were reconstructed using an ordered subset expectation maximization algorithm with weighted attenuation and smoothed with a 4‐mm Gaussian kernel with scatter correction.

Distribution volume ratio (DVR) was generated with Logan graphical analysis on PiB frames over 35 to 90 minutes post‐injection and normalized using a cerebellar gray matter reference region.^45^, ^46^ Global cortical PiB DVR was calculated using FreeSurfer‐derived cortical regions of interest (ROIs),^39^, ^47^ and Aβ positivity determined using a global PiB DVR threshold of 1.065.^42^ Centiloid (CL) values were calculated using a conversion equation employed previously in our laboratory^48^ and developed for our processing pipeline: CL = (DVR × 142.73) – 141.99.

For FTP‐PET images, participants were injected with 10 mCi of tracer and scanned from 80 to 100 minutes post‐injection, usually on the same day as the PiB‐PET scan. CT scans were used for attenuation correction. FTP‐PET images were reconstructed using an ordered subset expectation maximization algorithm with scatter correction and smoothed with a 4‐mm Gaussian kernel.

To create FTP standardized uptake value ratio (SUVR) images, the mean tracer uptake 80 to 100 minutes post‐injection was normalized to the inferior cerebellar gray matter reference region.^49^ Geometric transfer matrix partial volume correction (PVC) on the Desikan‐Killiany FreeSurfer‐derived ROIs was used for FTP data processing to account for partial volume effects.^50^, ^51^ The Desikan‐Killiany atlas was used to define ROIs of the entorhinal cortex (EC) and inferior temporal (IT) cortex, which were used to explore differences between SA and TA in FTP uptake. These regions were chosen because tau accumulation has been shown to start focally in the EC, and the IT was chosen as an early‐stage tau deposition region outside the medial temporal lobe (MTL).^52^, ^53^, ^54^, ^55^

### 2.7 Statistical analyses

Statistical analyses were performed using MATLAB R2021a, RStudio version 1.4.1717, and FreeSurfer version 5.3. Between‐group differences (training cohort vs test cohort, and SA groups vs TA) in demographic and clinical features were investigated using Welch two‐sample *t*‐test and Cohen's d effect size for continuous variables and chi‐square test and Cramér's V for categorical variables. Agreement between SA definitions were assessed using Cohen's kappa statistic coefficients (κ).

Differences between SA groups and TA in cortical thickness were assessed with vertex‐wise general linear models (GLMs) in FreeSurfer including age, sex, and years of education as covariates of no interest. First, we used a false discovery rate (FDR) threshold of *q* = 0.05 to correct for multiple comparisons.^56^ If no result survived correction for multiple comparisons, a threshold of *p* < 0.001 uncorrected was applied. Vertex‐wise associations between cortical thickness and CAG, EM, and NM composite scores, and CVLT LDFR were also explored controlling for sex and years of education. Age was additionally included as a covariate of no interest in CVLT‐related analysis because it was the only measure that was not age adjusted. Linear relationships are displayed vertex‐wise at *p*‐values adjusted for multiple comparisons (FDR *q* = 0.05).

A GLM was used to explore differences between SA and TA in TIV‐adjusted hippocampal volume, global PiB DVR, and FTP SUVR in the EC and IT ROIs separately for each SA group. Specifically, we performed analyses of covariance (ANCOVA) controlling for age, sex, and years of education. We repeated the analyses including days between cognitive session and MRI/PET scan as an additional covariate of no interest. Partial eta squared (partial η^2^) was used as a measure of the effect size for between‐group differences (small = 0.01, medium = 0.06, large = 0.14). Significance level was set at *p* < 0.05.

## 3 RESULTS

### 3.1 Cognitive age model accurately predicts age from neuropsychological tests

To estimate cognitive‐predicted age we used PLSr, which performs latent space modeling, deriving latent features that have maximum covariance with the response variable. We trained the CA model using an independent data set (BACS training cohort) than the data set used to associate with imaging variables (BACS test cohort). These training and testing data sets were generally well matched (Table 1); however, there was a significantly smaller proportion of female participants and greater rates and duration of follow‐up for the testing set. The percentage of variance explained by each component in the trained CA model is described in the Supplementary results, which suggest that only components 1 and 2 are well associated with age (explaining 33% and 13% of variance, respectively). Figure S2 shows the contribution of each predictor to the first two components.

**TABLE 1:** Cognitive age model cohort characteristics.

Characteristic	Training cohort (*n* = 293)	Test cohort (*n* = 238)	*p*‐value
Age	73.48 (8.15)	74.00 (7.05)	0.43
Sex, female, *n* (%)	192 (66)	133 (56)	0.03
Education, years^a^	16.80 (2.18)	16.81 (2.15)	0.96
History of hypertension, Y, *n* (%)^b^	100 (40)	91 (38)	0.73
History of diabetes, Y, *n* (%)^b^	22 (9)	26 (11)	0.52
History of heart disease, Y, *n* (%)^b^	31 (12)	35 (15)	0.54
Repeated cognitive assessments, *n* (%)	92 (31)	205 (86)	<0.001
Years of cognitive follow‐up	0.97 (1.86)	5.29 (3.98)	<0.001
Race/ethnicity			
Asian, n (%)	10 (3)	16 (7)^d^	–
Black or African American, *n* (%)	12 (4)	6 (3)^e^	–
Native Hawaiian or other Pacific Islander, *n* (%)	–	3 (1)	–
Hispanic or Latino, *n* (%)	7 (2)^c^	9 (4)^f^	–
White, *n* (%)	175 (60)	213 (89)	–
Unknown, *n* (%)	95 (32)	–	

*Note*: Values represent either mean (SD) or n (%). Differences between groups were investigated using Welch two‐sample *t*‐test for continuous variables and chi‐square test for categorical variables. Abbreviations: Y, yes.

^a^ Missing data for two participants in the training cohort.

^b^ Missing data for 44 participants in the training cohort.

^c^ Including Hispanic or Latino and White (*n* = 4), Hispanic or Latino and Asian (*n* = 1), Hispanic or Latino and Black (*n* = 1).

^d^ Including Asian and White (*n* = 2).

^e^ Including Black and White (*n* = 2).

^f^ Including Hispanic or Latino and Asian (*n* = 1), Hispanic or Latino and White (*n* = 6), Hispanic or Latino and Native Hawaiian or other Pacific Islander (*n* = 2).

**FIGURE 1:**
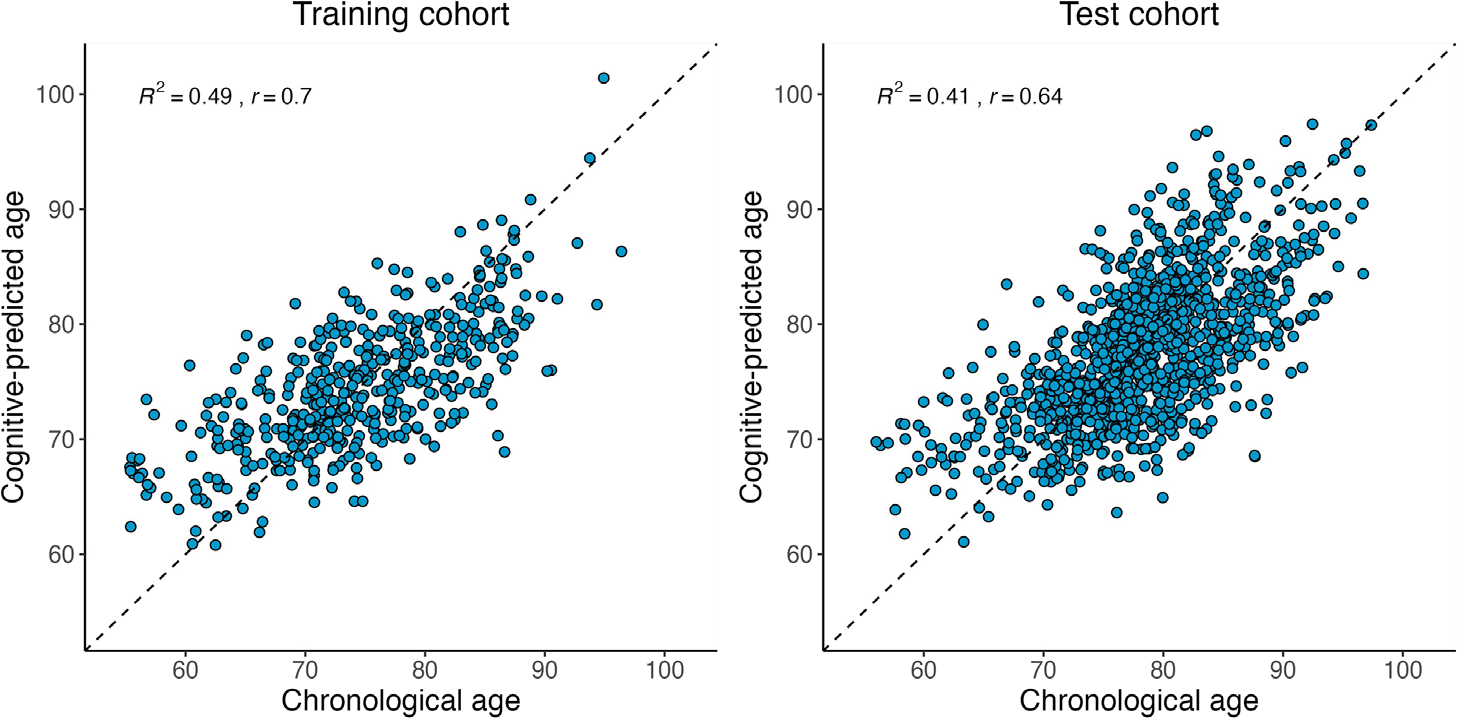
Cognitive‐predicted age from the partial least squared (PLS) regression model. Scatterplots showing chronological age by cognitive‐predicted age before age‐bias correction in the training (left) and test (right) cohorts. The dashed lines are lines of identity (x = y) where cognitive‐predicted age = chronological age. *R* ^2^ values refers to the total variance explained, and *r*‐values are the Pearson correlation coefficients of cognitive‐predicted age with chronological age.

Applying the parameters learned from the trained model to predict age in the independent test cohort (*n* = 1141 sessions), the model accurately predicted age with a MAE of 4.36, explaining 41% of the variance in chronological age (Figure 1). This value was similar to the variance explained in the training sample (*R* ^2^ = 0.49). For each session, CAG scores were obtained by subtracting chronological age at each session from the corresponding predicted age and correcting for known biases.^33^, ^34^, ^35^ Figure S3 shows the association between chronological age and CAG estimates/cognitive age before and after applying the age‐bias correction proposed by Beheshti et al.^36^ in the training and test cohort to account for a frequently observed bias in age prediction resulting in an overestimated age for younger adults and underestimated age for older adults.^33^, ^34^, ^35^ Overall, these results validated our modeling approach to predict age using cognitive data.

### 3.2 Four definitions of SA (70+ years old)

**FIGURE 2:**
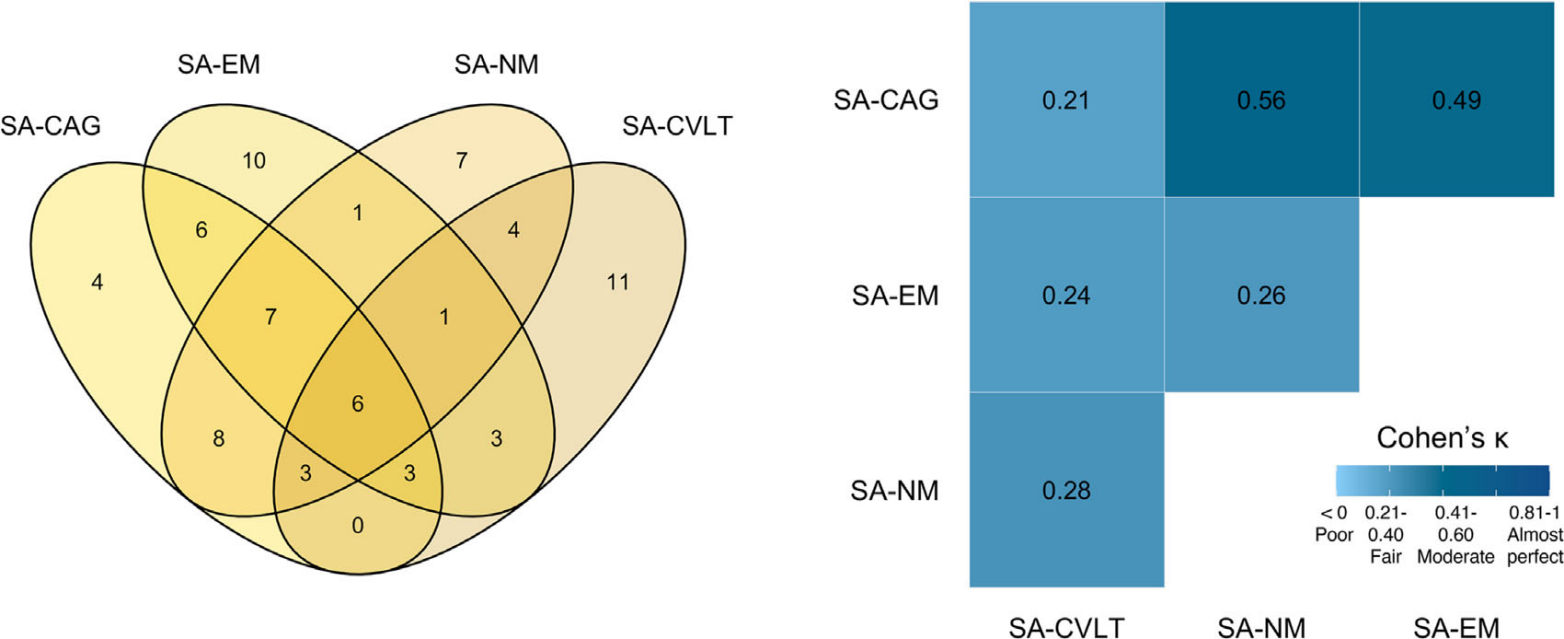
Number of overlapping participants included in the successful cognitive aging (SA) groups using different definitions (left), and Cohen's κ values indicating the strength of agreement between definitions (right). The strength of agreement given by the Cohen's κ coefficients was interpreted as poor (<0), slight (0–0.20), fair (0.21–0.40), moderate (0.41–0.60), substantial (0.61–0.80), and almost perfect (0.81–1).^57^

Using a subset of BACS individuals aged 70 and older from the test cohort (*n* = 184), we explored the number of participants identified as exceptional cognitive performers, herein called SA, by different new and previously accepted definitions. Participants were identified as SA based on (1) ≤20th percentile as CAG (SA‐CAG, *n* = 37); (2) ≥80th percentile of age‐adjusted EM composite (SA‐EM, *n* = 37); (3) ≥80th percentile of age‐adjusted NM cognition composite cutoff score (SA‐NM, *n* = 37); and (4) performance comparable to young adults on the CVLT LDFR (score of 14 or above; max score = 16) (SA‐CVLT, *n* = 31). All individuals in the cohort not meeting any of the four SA criteria were defined as TA (*n* = 110). Cohen's kappa statistics were used to explore the strength of agreement between definitions. The number of overlapping participants defined as SA by different definitions and Cohen's κ coefficients, displayed in Figure 2, showed only a fair to moderate strength of agreement between definitions, with only 6 of 74 SA participants defined as SA by all definitions. The strength of agreement given by the Cohen's κ coefficients was interpreted as poor (<0), slight (0–0.20), fair (0.21–0.40), moderate (0.41–0.60), substantial (0.61–0.80), and almost perfect (0.81–1).^57^ The CAG‐based definition presented the best overlap with other definitions, with the lowest number of participants defined as SA‐CAG only (*n* = 4), as opposed to other definitions (only SA‐EM = 10; only SA‐NM = 7; only SA‐CVLT = 11). When we repeated the analyses with the same number of participants within each SA group to match the SA‐CVLT number of participants (*n* = 31), the results were similar (Cohen's κ coefficients: SA‐CAG and SA‐EM, κ = 0.42; SA‐CAG and SA‐NM, κ = 0.53; SA‐CAG and SA‐CVLT, κ = 0.22; SA‐EM and SA‐NM, κ = 0.30; SA‐EM and SA‐CVLT, κ = 0.26; and SA‐NM and SA‐CVLT, κ = 0.30).

### 3.3 SA and TA cohort characteristics

Cohort characteristics for SA and TA are summarized in Table 2. Successful cognitive aging defined using CAG, EM, NM, or CVLT were also combined in a single group of individuals meeting any SA criterion (SA‐ALL, *n* = 74) and compared with TA. Each SA group was compared with the same group of individuals defined as TA (all individuals in the cohort not meeting any of the four SA criteria, *n* = 110). A higher percentage of female individuals was found in SA‐CVLT (𝜒^2^(1) = 10, *p* = 0.002, V = 0.27), and SA‐ALL (𝜒^2^(1) = 4.90, *p* = 0.03, V = 0.16) compared to TA. Moreover, significantly higher years of education were reported in SA‐ALL (t(173.93) = 3.3, *p* = 0.001, d = 0.48), SA‐EM (t(85.30) = 2.80, *p* = 0.02, d = 0.46), SA‐NM (t(66.84) = 2.53, *p* = 0.01, d = 0.46), and SA‐CVLT (t(53.28) = 3.3, *p* = 0.002, d = 0.63), but not SA‐CAG (t(72.34) = 1.64, *p* = 0.11, d = 0.29). SA‐CVLT had a higher proportion of participants with self‐reported family history of dementia (𝜒^2^(1) = 5.83, *p* = 0.02, V = 0.21). No differences between any SA group and TA were found in age, BMI, history of hypertension, or MMSE and GDS scores. Finally, we were interested in the effect of the *APOE* ε4 allele, the major genetic risk factor for sporadic AD, and the APOE ε2 allele for its protective effect.^58^, ^59^ Participants were grouped as APOE ε2 carriers (ε2/ε3, *n* = 16), APOE ε3 homozygotes (ε3/ε3, *n* = 116), or APOE ε4 carriers (ε3/ε4, *n* = 42; ε4/ε4, *n* = 1). No APOE ε2/ε2 homozygotes were identified, whereas APOE ε2/ε4 heterozygotes (*n* = 3) were excluded from these analyses. No differences in *APOE* genotype were found across SA definitions compared with TA. Neuropsychological tests are summarized in Table S1.

**TABLE 2 alz13438-tbl-0002:** Successful and typical aging cohort characteristics.

							*p*‐value, effect size				
Characteristic	TA (*n* = 110)	SA‐ALL (*n* = 74)	SA‐CAG (*n* = 37)	SA‐EM (*n* = 37)	SA‐NM (*n* = 37)	SA‐CVLT (*n* = 31)	SA‐ALL vs TA	SA‐CAG vs. TA	SA‐EM vs. TA	SA‐NM vs. TA	SA‐CVLT vs. TA
Age	75.8 (5.03)	76.3 (5.49)	77.4 (5.23)	77.1 (5.59)	76.8 (5.51)	74.4 (4.44)	0.52, d = 0.10	0.11, d = 0.31	0.20, d = 0.26	0.30, d = 0.21	0.15, d = 0.28
Sex, female, *n* (%)	55 (50)	50 (68)	23 (62)	20 (54)	24 (65)	26 (84)	0.03, V = 0.16	0.27, V = 0.09	0.81, V = 0.02	0.17, V = 0.11	0.002, V = 0.27
Education, years	16.3 (2.14)	17.3 (1.78)	16.9 (1.82)	17.2 (1.55)	17.3 (1.97)	17.6 (1.91)	0.001, d = 0.48	0.11, d = 0.29	0.02, d = 0.46	0.01, d = 0.46	0.002, d = 0.63
BMI^a^	26.4 (3.98)	26.8 (5.00)	27.3 (4.66)	26.2 (4.48)	26.6 (4.91)	27.4 (5.61)	0.61, d = 0.08	0.29, d = 0.22	0.83, d = 0.04	0.81, d = 0.05	0.35, d = 0.23
History of hypertension, Y, *n* (%)	50 (45)	24 (32)	14 (38)	14 (38)	13 (35)	8 (26)	0.11, V = 0.12	0.54, V = 0.05	0.54, V = 0.05	0.37, V = 0.07	0.08, V = 0.15
Family history of dementia, Y,n (%)^b^	29 (28)	23 (32)	11 (30)	14 (38)	14 (38)	16 (53)	0.69, V = 0.03	0.97, V = 0.003	0.34, V = 0.08	0.34, V = 0.08	0.02, V = 0.21
MMSE	28.7 (1.25)	28.8 (1.39)	28.8 (1.34)	28.9 (1.28)	28.9 (1.22)	28.8 (1.39)	0.63, d = 0.07	0.82, d = 0.04	0.43, d = 0.15	0.48, d = 0.13	0.70, d = 0.09
GDS	3.49 (2.74)	3.03 (2.99)	3.16 (2.70)	2.97 (3.06)	2.86 (3.02)	2.84 (2.41)	0.29, d = 0.16	0.53, d = 0.12	0.36, d = 0.18	0.27, d = 0.22	0.20, d = 0.24
*APOE* ε2/ε3/ε4 (%)^c^	8/68/24	11/64/25	11/60/30	14/64/22	8/59/32	10/60/30	0.73, V = 0.06	0.66, V = 0.08	0.55, V = 0.09	0.61, V = 0.08	0.72, V = 0.07
PiB DVR	1.14 (0.22)	1.10 (0.17)	1.12 (0.18)	1.10 (0.16)	1.12 (0.18)	1.13 (0.19)	0.26, d = 0.16	0.71, d = 0.06	0.27, d = 0.18	0.70, d = 0.07	0.81, d = 0.04
PiB+, *n* (%)	46 (42)	30 (41)	15 (41)	14 (38)	17 (46)	13 (42)	0.98, V = 0.001	1.00, V = 0.00	0.82, V = 0.02	0.81, V = 0.02	1.00, V = 0.00
FTP scan available, *n*	73	41	16	25	14	21	–	–	–	–	–
Race/ethnicity							–	–	–	–	–
Asian, *n* (%)	8 (7)^d^	2 (3)	2 (5)	–	1 (3)	–	–	–	–	–	–
Black or African American, *n* (%)	4 (4)^e^	1 (1)^e^	–	1 (3)^e^	–	–	–	–	–	–	–
Native Hawaiian or other Pacific Islander, *n* (%)	3 (3)	–	–	–	–	–	–	–	–	–	–
Hispanic or Latino, *n* (%)	4 (4)^f^	2 (3)^g^	–	–	–	2 (6)^g^	–	–	–	–	–
White, *n* (%)	95 (86)	71 (96)	35 (95)	36 (97)	36 (97)	31 (100)	–	–	–	–	–

*Note*: Values represent either mean (SD) or n (%). Differences between typical aging (TA) and each successful cognitive aging (SA) group were assessed using Welch two‐sample *t*‐test (Cohen's *d* as effect size) for continuous variables and chi‐square test (Cramér's V as effect size) for categorical variables.

Abbreviations: BMI, body mass index; CAG, cognitive age gap; CVLT, California Verbal Learning Test; EM, episodic memory; GDS, Geriatric Depression Scale; MMSE, Mini‐mental State Examination; NM, non‐memory cognition; PiB DVR, Pittsburgh compound B distribution volume ratio; SA, successful cognitive aging; TA, typical aging; Y, yes.

^a^ Missing data for five TA individuals.

^b^ Missing data for three TA individuals.

^c^ Missing data for five TA and one SA‐CAG/SA‐ALL (participants were grouped as *APOE* ε2 carriers (ε2/ε3), *APOE* ε3 homozygotes (ε3/ε3), or *APOE* ε4 carriers (ε3/ε4 and ε4/ε4, *n* = 1); no *APOE* ε2/ε2 homozygotes were identified, whereas *APOE* ε2/ε4 heterozygotes (*n* = 3) were excluded from these analyses.

^d^ Including Asian and White (*n* = 1).

^e^ Including Black and White (*n* = 1).

^f^ Including Hispanic or Latino and White (*n* = 2), Hispanic or Latino and Native Hawaiian or other Pacific Islander (*n* = 2).

^g^ Including Hispanic or Latino and White (*n* = 2).

### 3.4 Greater medial prefrontal and temporal thickness, and greater hippocampal volume in successful cognitive aging

**FIGURE 3:**
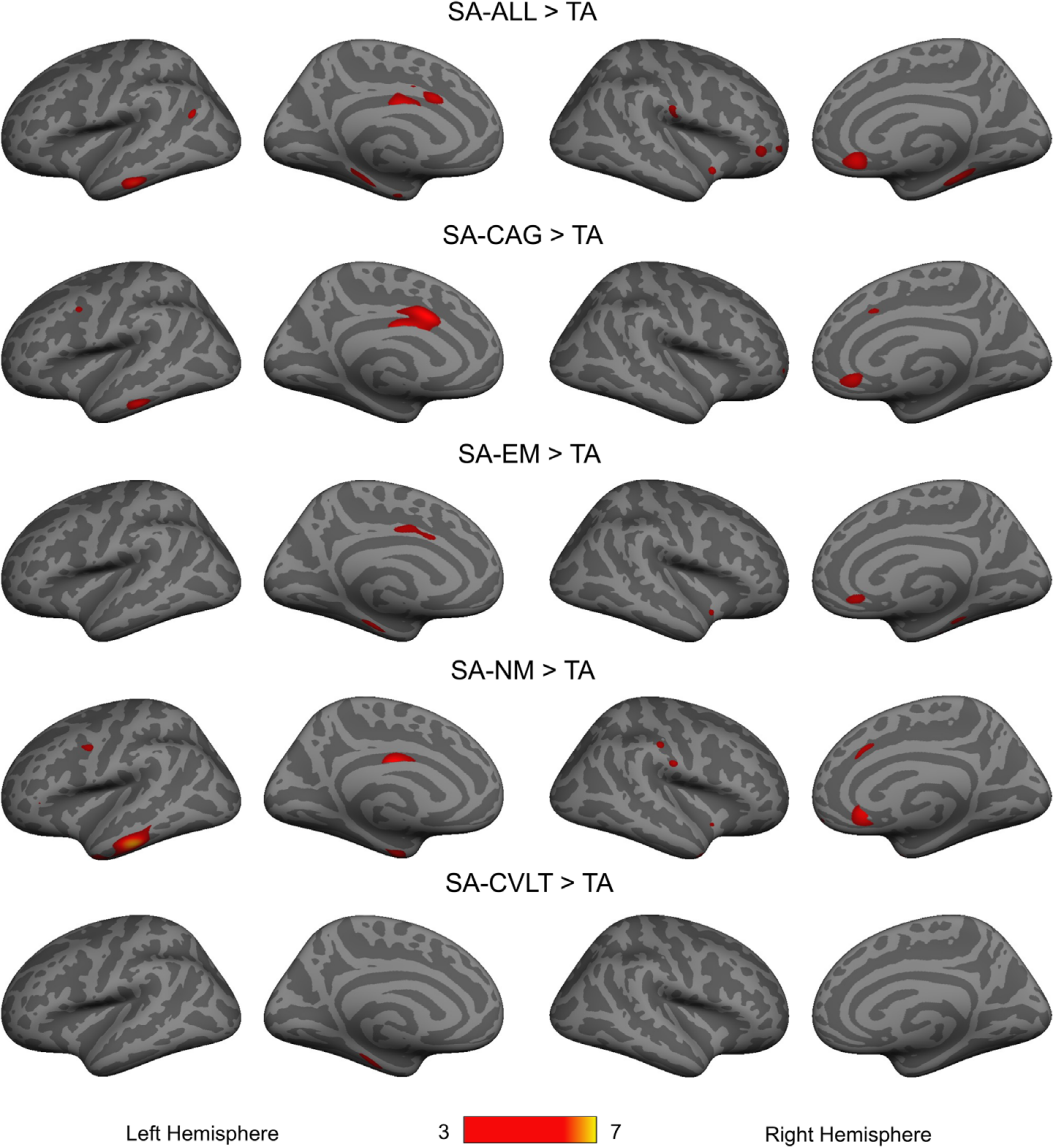
Whole‐brain vertex‐wise cortical thickness analyses revealing regions of significantly thicker cortex in successful cognitive aging (SA) compared to typical aging (TA) accounting for age, sex, and years of education. Significance threshold was set at *p* < 0.001 uncorrected. Color bar indicates logarithmic scale of *p*‐values (–log10). No region was found as significantly thicker in TA compared with SA.

Vertex‐wise cortical thickness analyses in FreeSurfer revealed regions of greater cortical thickness in SA groups compared to TA[Table alz13438-tbl-0002] (*p* < 0.001 uncorrected, Figure 3). All results were adjusted for age, sex, and years of education. For the SA‐ALL group, the following regions were thicker compared with TA: left anterior midcingulate cortex (aMCC), also known as dorsal anterior cingulate cortex, left posterior midcingulate cortex (pMCC), left middle and inferior temporal gyri, right rostral anterior cingulate cortex (rACC) extending to the medial orbitofrontal cortex (mOFC), and MTL, mostly in the bilateral parahippocampal gyrus and left EC. All other SA groups except SA‐CVLT showed thicker left MCC, right rACC/mOFC, and ACC. SA‐EM, SA‐NM, and SA‐CVLT had thicker regions in the MTL. A cluster in the lateral temporal lobe was thicker in SA‐CAG and SA‐NM compared with TA. It is important to note that there were no regions in the brain where cortex was thicker in TA compared with SA, regardless of definition. When education was removed from the vertex‐wise analyses, the findings were consistent.

**FIGURE 4:**
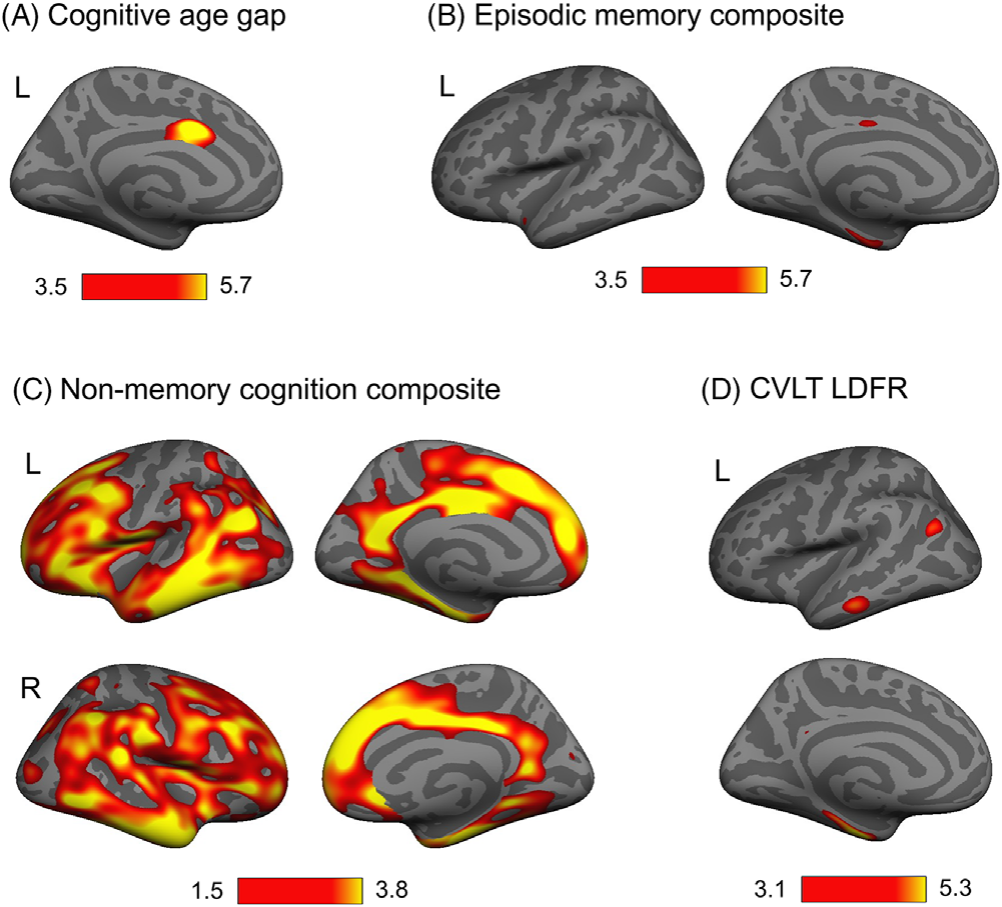
Whole‐brain vertex‐wise cortical thickness analyses revealing regions of significant association with: (A) cognitive age gap (CAG; negative association), (B) age‐adjusted episodic memory composite (positive association), (C) age‐adjusted non‐memory cognition composite (positive association), and (D) California Verbal Learning Test Long Delay Free Recall (CVLT LDFR; positive association) in the whole sample, including all successful cognitive aging (SA) groups and typical aging (TA). Significance threshold was set at *q* = 0.05 false discovery rate (FDR) corrected for multiple comparisons. Color bars indicate the general linear model (GLM)–specific FDR‐adjusted logarithmic scale of *p*‐values (–log10). Sex and years of education were included as covariates of no interest in all analyses, and age was additionally included in CVLT‐related analyses.

A series of GLMs were run in FreeSurfer to test the continuous relationship between cognitive measures and cortical thickness in the whole sample (including all SA groups and TA). Sex and years of education were included as covariates of no interest; in addition, age was included in CVLT‐related analyses. GLMs revealed a negative association between CAG scores and the left aMCC/pMCC (Figure 4A, *p* < 0.05 FDR corrected). EM composite scores were positively associated with cortical thickness in the left MTL (parahippocampal cortex and EC) and pMCC; CVLT LDFR was positively associated with cortical thickness in the left parahippocampal, EC, middle and inferior temporal, and inferior parietal cortices. The NM composite score was positively associated with widespread thickness in cortical brain regions (Figure 4B‐D, *p* < 0.05 FDR corrected).

**FIGURE 5:**
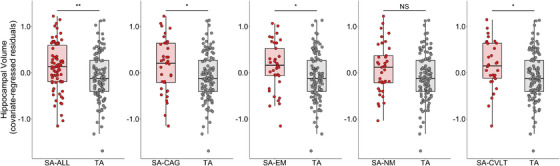
Compared to TA greater TIV‐adjusted hippocampal volume was found in SA‐ALL, SA‐CAG, SA‐EM, and SA‐CVLT controlling for age, sex, years of education. CAG, cognitive age gap; CVLT, California Verbal Learning Test; EM, episodic memory; NM, non‐memory cognition; SA, successful cognitive aging; TA, typical aging; TIV, total intracranial volume. *p < 0.05, **p < 0.01.

Hippocampal volumes were extracted using the FreeSurfer volumetric segmentation^41^ and differences between SA groups and TA were explored using ANCOVA models including age, sex, and years of education as covariates of no interest. The following model was examined separately for each SA group: hippocampal volume ∼ SA Group + Age + Sex + years of education. Significant differences were found between TA and SA‐ALL (F(1, 179) = 8.43, *p* = 0.004, partial η^2^ = 0.04), SA‐CAG (F(1, 142) = 6.05, *p* = 0.02, partial η^2^ = 0.04), SA‐EM (F(1, 142) = 4.49, *p* = 0.04, partial η^2^ = 0.03), and SA‐CVLT (F(1, 136) = 5.94, *p* = 0.02, partial η^2^ = 0.04), but there were no differences between TA and SA‐NM (F(1, 142) = 3.24, *p* = 0.07, partial η^2^ = 0.02) (Figure 5). Findings were replicated when years of education was removed from the models.

### 3.5 Lower entorhinal tau burden in successful cognitive aging, but no differences in global Aβ

We examined the relationship between successful aging and AD biomarkers, including PET measured PiB and FTP to measure Aβ and tau burden, respectively. We explored differences between SA and TA in FTP uptake in the EC as an early region of tau accumulation, and in the IT cortex as an early stage tau deposition region outside the MTL.^52^, ^53^, ^54^, ^55^ We also investigated FTP uptake in ACC/MCC ROIs, since previous neuropathological evidence has shown lower NFTs in the rACC and aMCC regions in SuperAgers compared with age‐matched controls.^25^

Differences between SA groups and TA in global PiB DVR and PVC FTP uptake in each ROI were explored using ANCOVA models including age, sex, and years of education as covariates of no interest. The following models were performed separately for each SA group: global PiB DVR or ROI FTP uptake ∼ SA Group + Age + Sex + years of education.

No differences were found between SA groups and TA in global PiB DVR and in the proportion of PiB‐positive (PiB DVR >1.065) participants (*p* > 0.05, Table 2). No significant effect of SA on global PiB DVR was present after controlling for covariates (SA‐ALL, (F(1, 179) = 1.04, *p* = 0.31, partial η2 = 0.006), SA‐CAG (F(1, 142) = 0.14, *p* = 0.71, partial η2 = 0.001), SA‐EM (F(1, 142) = 0.46, *p* = 0.50, partial η2 = 0.003), SA‐NM (F(1, 142) = 0.05, *p* = 0.82, partial η2 = 0.0004), and SA‐CVLT (F(1, 136) = 0.22, *p* = 0.64, partial η^2^ = 0.002)).

**FIGURE 6:**
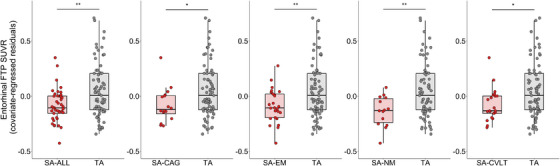
Compared to TA, lower entorhinal FTP PVC SUVR was found in all SA groups controlling for age, sex, years of education. CAG, cognitive age gap; CVLT, California Verbal Learning Test; EM, episodic memory; FTP, flortaucipir; NM, non‐memory cognition; PVC, partial volume correction; SA, successful cognitive aging; SUVR, standardized uptake value ratio; TA, typical aging. *p < 0.05, **p < 0.01.

Significant differences in EC FTP uptake were found between TA and SA‐ALL (F(1, 109) = 10.01, *p* = 0.002, partial η^2^ = 0.08), SA‐CAG (F(1, 84) = 4.06, *p* = 0.047, partial η^2^ = 0.05), SA‐EM (F(1, 93) = 7.17, *p* = 0.009, partial η^2^ = 0.07), SA‐NM (F(1, 82) = 7.69, *p* = 0.007, partial η^2^ = 0.09), and SA‐CVLT (F(1, 89) = 4.46, *p* = 0.04, partial η^2^ = 0.05) (Figure 6). The results remained similar when we included days between cognitive session and PET scan as a covariate of no interest in addition to age, sex, and years of education (see Supplementary results). Moreover, findings were replicated when years of education was removed from the models. Next, we repeated the model including global PIB DVR as a covariate of no interest: EC FTP uptake ∼ SA Group + Age + Sex + years of education + PiB DVR. There was a significant effect of SA group on EC FTP uptake when controlling for PiB DVR for SA‐ALL (F(1, 107) = 6.71, *p* = 0.01, partial η^2^ = 0.06), SA‐EM (F(1, 91) = 6.14, *p* = 0.02, partial η^2^ = 0.06), and SA‐NM (F(1, 80) = 6.43, *p* = 0.01, partial η^2^ = 0.07), but not SA‐CAG (F(1, 82) = 2.66, *p* = 0.11, partial η^2^ = 0.03) and SA‐CVLT (F(1, 87) = 3.47, *p* = 0.07, partial η^2^ = 0.04).

Differences between SA and TA in IT FTP uptake were significant only in SA‐ALL (F(1, 109) = 4.20, *p* = 0.04, partial η^2^ = 0.04), with no significant effects when SA was defined as SA‐CAG (F(1, 84) = 3.49, *p* = 0.07, partial η^2^ = 0.04), SA‐EM (F(1, 93) = 3.31, *p* = 0.07, partial η^2^ = 0.03), SA‐NM (F(1, 82) = 2.45, *p* = 0.12, partial η^2^ = 0.03), and SA‐CVLT (F(1, 89) = 1.93, *p* = 0.17, partial η^2^ = 0.02). The results remained unaltered when we included days between cognitive session and PET scan as covariate of no interest in addition to age, sex, and years of education (see Supplementary results). When years of education was removed from the model, we found a significant effect of SA group on IT FTP when all SA were grouped together (SA‐ALL: F(1, 110) = 4.98, *p* = 0.03, partial η^2^ = 0.04), and SA‐EM (F(1, 94) = 4.29, *p* = 0.04, partial η^2^ = 0.04), but no effect when SA was defined as SA‐CAG, SA‐NM, and SA‐CVLT. Finally, no significant differences were found between SA groups and TA in rACC, aMCC, and pMCC FTP uptake.

## 4 DISCUSSION

Despite imperfect overlap between the four SA groups, our findings suggest common brain features across definitions. Except for SA‐CVLT, all SA groups presented greater cortical thickness in the aMCC/pMCC, rACC/mOFC compared to TA. SA also had regions of thicker cortex in the MTL (SA‐EM, SA‐NM, and SA‐CVLT) and lateral temporal regions (SA‐CAG and SA‐NM). In addition, SA‐CAG, SA‐EM, and SA‐NM had greater hippocampal volume and all SA groups had lower EC tau burden compared to TA. Overall, these findings suggest that a feature of SA, regardless of its exact definition, may be resistance to tau pathology, whereas greater thickness in aMCC, rACC/mOFC, and MTL may be interpreted as greater brain reserve.

The first goal of this study was to develop a new measure to define SA using a CA prediction model. Our findings suggest that we can reliably predict age from neuropsychological tests and use these age estimates to calculate biologically meaningful CAG scores. We also defined SA based on EM and NM cognition composite scores as well as performance on the CVLT LDFR. Our findings revealed that different definitions of SA identified only partially overlapping groups of older adults, highlighting the heterogeneity of the successful aging concept and its definition. Moderate strength of agreement was found between SA‐CAG and SA‐NM/EM definitions, but only fair agreement was shown between the others. The imperfect overlap of SA groups highlights the importance of considering different SA definitions and approaches when interpreting results from studies on SA.

Previous studies have defined SA predominantly as older adults with exceptional memory performance, mostly due to the vulnerability of memory abilities to both aging and AD.^7^ There may be conceptual and neurobiological distinctions associated with the use of domain‐specific SA definitions. Hence, in the present study, we decided to include both memory and non‐memory cognition definitions to investigate potential divergent neurobiological substrates. Moreover, substantial data indicate that women have better performance on verbal memory tests,^60^ which is also reflected in our findings. For example, SA‐CVLT had a significantly higher percentage of women, but no sex differences were found between SA‐CAG and TA. Our novel definition based on a cognitive age model aimed at capturing patterns of holistic cognitive aging that may not be easily detectable by single or composite measures of cognitive performance. SA‐CAG showed the highest overlap with the other definitions, suggesting that it may be a more comprehensive measure capturing multiple aspects of SA. Moreover, SA‐CAG was the only SA group that did not differ from TA in years of education, suggesting that this definition may be less dependent on educational attainment, while it appeared to be a feature of the other definitions. For this reason, we repeated the analyses both with and without education in our models, and the results remained consistent, indicating that education, overall, had no significant impact on our outcome measures.

To address our second aim, we explored differences between TA and each SA group in brain features. We found regions of greater cortical thickness in the aMCC/pMCC, rACC/mOFC in SA using most definitions, compared with TA, confirming previous results in successful memory aging.^4^, ^14^, ^22^ The observation that the SA‐CVLT group showed thicker cortex limited to the MTL may reflect the episodic memory–predominant definition of this group. The consistency of thicker aMCC/pMCC cortex across SA definitions was striking. Furthermore, when we explored the continuous relationship between cognitive scores and cortical thickness, we found a negative association between continuous CAG and the aMCC/pMCC thickness (i.e., greater thickness for younger predicted cognitive age). This is consistent with our interpretation that the CAG captures cognitive performance well across multiple definitions of SA and may be more sensitive to superior cognitive performance rather than pathological cognitive impairment. The strong relationship between continuous CAG and thicker aMCC/pMCC suggests the CAG is a promising continuous measure of normal cognitive aging that can be used as an alternative approach to the dichotomization into two separate groups (i.e., TA vs SA).

Thickness in the ACC/MCC and its relationship to SA is particularly interesting due to its unique neurobiology. Evidence from studies of SuperAging demonstrate greater cortical thickness of this brain region in SA, even with different specific comparator groups.^4^, ^25^ This region is also one of several, including the orbitofrontal cortex and frontal regions of the insula, that contain relatively high densities of Von Economo neurons (VENs). These unusual spindle‐shaped neurons have unclear functional significance; are seen in humans, great apes, and cetaceans; and are selectively vulnerable to neurodegeneration in frontotemporal dementia in humans.^61^, ^62^ Brain regions with a high density of VENs have been implicated in a variety of neuropsychiatric disorders involving emotional‐social functions, and also comprise the main hubs of the salience network.^63^, ^64^ Postmortem studies of SuperAgers have shown that these individuals have substantially higher numbers of VENs in the aMCC compared to age‐matched cognitively normal individuals as well as younger controls.^65^ Lower levels of tau pathology in rACC and aMCC have been shown previously in SuperAgers compared to both age‐matched controls and cases of mild cognitive impairment (MCI).^25^ In our study, however, we found no differences in FTP uptake in ACC/MCC regions, which could be due to lower levels of tau burden in both SA and TA groups in our sample in these regions.

Taken together, the structural MRI findings in this cohort, along with other histological studies, suggest that the MCC is an important region for optimal brain aging outcomes. Greater cortical thickness in this brain region is consistent across studies of SuperAgers^4^, ^25^ and other definitions of SA,^14^, ^22^ and metabolic preservation in this region in cognitively normal older people also behaves as a signature of brain resilience.^66^ Other studies have shown hypertrophy of cortical neurons in the ACC in asymptomatic individuals with AD pathology in comparison with age‐matched controls, MCI, and AD patients, which could be interpreted as either an early response to AD pathology or a compensatory mechanism.^67^, ^68^, ^69^ It is not clear whether these brain regions provide support to successful aging outcomes through a dynamic or adaptive process such as hypertrophy or even neurogenesis, or whether they reflect lifelong advantages related to genetic or early life environmental effects. Regardless, together these findings provide strong evidence for the importance of the ACC and MCC in maintaining superior cognition at an older age, regardless of SA definition and methodological differences across studies.

We also found evidence that preserved integrity of the MTL was a feature of SA. Greater hippocampal volume has been related previously to SA.^8^, ^14^, ^22^ This may be interpreted as greater brain reserve, so that interindividual neurobiological differences may allow SA to overcome the effects of brain aging.^70^ However, further evidence from longitudinal studies is needed to clarify the role of individual brain morphological changes over time in SA. The conjoint findings of volume preservation in MTL/hippocampus and reduced deposition of pathological tau raise the possibility that these findings are related. The accumulation of hyperphosphorylated tau in intracellular NFTs, a hallmark of AD, occurs in a pattern of topographic distribution similar to that of brain atrophy, can also be found in cognitively normal older adults, and is associated with cognition.^44^, ^71^, ^72^, ^73^ There is also strong evidence of relationships between tau accumulation and regional MTL atrophy in cognitively normal older people.^74^, ^75^ Based on this evidence, it is possible that the finding of larger hippocampal volumes and thicker MTL cortex in the SA sample could reflect the reduced tau pathology, lifelong or early life‐reserve factors, a dynamic response to pathology, or a combination of these factors.

Lower tau in our SA participants was seen despite comparable levels of Aβ. The mean DVR value measured with PiB‐PET in the TA group was 21 CL, whereas the mean values in the SA groups ranged from 15 to 19 CL. It is difficult to know whether these small differences are related to differences in EC tau burden. SA individuals may be resistant to EC tau deposition, consistent with previous neuropathological reports on SuperAging,^6^ and this may underlie, at least in part, their exceptional cognitive performance. Preliminary results from a small group of individuals from the Alzheimer's Disease Neuroimaging Initiative (ADNI) showed lower tau deposition in temporal and medial parietal lobe in SA compared with TA.^76^ It is important to note that our most robust findings on tau differences reflect tau deposition in the EC and not IT. These brain regions are different because EC tau is common in people without brain Aβ, whereas tau spread to IT usually reflects higher levels of Aβ.^44^, ^77^, ^78^, ^79^, ^80^, ^81^ These findings, along with the relatively small differences in brain Aβ, are consistent with a resistance of age‐related tau deposition in our SA participants rather than a resistance to AD pathology. Whether Aβ pathology plays a role in driving tau deposition in our SA group will require longitudinal observation.

Various limitations should be considered when interpreting the results of the present study. First, the BACS cohort is a fairly homogeneous and highly educated sample that is not fully representative of the diversity of typical cognitive aging. Second, there were some differences between the training and test cohorts for calculation of CAG, (i.e., presence of neuroimaging scans, number of follow‐up sessions, proportion of women), although all individuals underwent the same comprehensive screening process and received identical cognitive evaluations. Despite differences between the cohorts, when we applied the parameters learned from the trained model to predict age in the test sample, the model explained 41% of the variance in chronological age, which was very similar to the 49% variance explained in the training sample. This confirms the validity of our modeling approach by demonstrating that the model is robust to differences in training and test samples. Another limitation is that our methods reflect numerous choices of thresholds that are likely to affect our results. This includes the age threshold, the threshold for composite score definitions of SA or CVLT performance, and thresholds for statistical significance in a variety of analyses. Finally, this is a cross‐sectional investigation, and it is increasingly apparent that cognitive aging requires longitudinal observation to fully comprehend the many associated phenomena.^17^

Our findings support the hypothesis that a combination of structural integrity and resistance to tau pathology may underlie SA regardless of its exact definition and promote effective cognitive functioning at an older age. It will be important in the future to investigate the relationship between both genetics and modifiable lifestyle factors related to SA and brain pathology. A better understanding of the neural features related to SA may lead to the identification of targets for new interventions aiming at promoting healthy aging. This becomes even more relevant in the light of increasing evidence suggesting the importance of interventions promoting brain health in helping mitigate cognitive decline.^82^

## AUTHOR CONTRIBUTIONS

Stefania Pezzoli, Theresa M. Harrison, and William J. Jagust conceptualized and designed the study. Stefania Pezzoli, Joseph Giorgio, Adam Martersteck, and Lindsey Dobyns contributed to the methodology and data analysis. Stefania Pezzoli and William J. Jagust wrote the manuscript and all authors contributed to the reviewing and editing the manuscript.

## CONFLICT OF INTEREST STATEMENT

The authors have no competing interests to declare. Author disclosures are available in the supporting information.

## CONSENT STATEMENT

All participants provided written, informed consent for their participation in this study.

## Supporting information

Supporting Information

Supporting Information
