# Supplementary material for: Successful cognitive aging is associated with thicker anterior cingulate cortex and lower tau deposition compared to typical aging
Source: Alzheimers Dement. 2023 Aug 24;20(1):341–55. doi: 10.1002/alz.13438 (PMC10916939; doi:10.1002/alz.13438)
Supplement: Supplementary file 1 — Supporting Information [file ALZ-20-341-s002.docx]

**Successful cognitive aging is associated with thicker anterior cingulate cortex and lower tau deposition compared to typical aging**

Stefania Pezzoli^1,2^, Joseph Giorgio^1,3^, Adam Martersteck^1^, Lindsey Dobyns^1^, Theresa M. Harrison^1^ and William J. Jagust^1,2^

*^1^ Helen Wills Neuroscience Institute, University of California, Berkeley, Berkeley, CA, 94720, USA*

*^2^ Lawrence Berkeley National Laboratory, Berkeley, CA, 94720, USA*

*^3^ University of Newcastle, Newcastle, NSW, 2305, Australia*

**Supplementary materials**

**Supplementary results**

*Cognitive age model: variance explained.* The percentage of variance explained by each component in the trained cognitive age model was as follows: 1) 33%, 2) 13%, 3) 2%, 4) 1%, 5) 0.1%, suggesting only the first two components are well associated to age. We assessed weight stability of each predictor by generating 1000 bootstrapped samples in the training dataset drawing with replacement. For component 1 we observed that all predictors significantly contributed to component 1, whereas for component 2, 10 out of 20 predictors significantly contributed (Supplementary Figure S2). Component 1 showed a strong contribution of the cognitive variables whereas component 2 had a more robust loading of session number, suggesting this component may account for potential practice effects.

*Successful aging: supplementary analyses.* We performed analyses accounting for time difference between cognitive session and MRI/FTP PET scan, measured in days, in addition to other covariates (age, sex, and years of education). Adding this additional covariate to the models did not change the results.

There was a significant effect of SA group on hippocampal volume when SA were grouped (SA-ALL, F(1, 178)=8.44, p=0.004, partial η2=0.05), when SA were defined as SA-CAG (F(1, 141)=5.93, p=0.02, partial η2=0.04), SA-EM (F(1, 141)=4.42, p=0.04, partial η2=0.03), SA-CVLT (F(1, 135)=5.65, p=0.02, partial η2=0.04), but not SA-NM (F(1, 141)=3.20, p=0.08, partial η2=0.02).

There was a significant effect of SA group on EC FTP uptake when SA were grouped (SA-ALL, F(1, 108)=9.61, p=0.002, partial η2=0.08), when SA were defined as SA-CAG (F(1, 83)=3.99, p=0.049, partial η2=0.05) and SA-EM (F(1, 92)=7.00, p=0.01, partial η2=0.07), SA-NM (F(1, 81)=7.58, p=0.007, partial η2=0.09), and SA-CVLT (F(1, 88)=4.32, p=0.04, partial η2=0.05).

We found a significant effect of SA group on IT FTP when SA were grouped (SA-ALL, F(1, 108)=4.21, p=0.04, partial η2=0.04), but no effect when SA were defined as SA-CAG (F(1, 83)=3.48, p=0.07, partial η2=0.04) and SA-EM (F(1, 92)=3.31, p=0.07, partial η2=0.03), SA-NM (F(1, 81)=2.43, p=0.12, partial η2=0.03), and SA-CVLT (F(1, 88)=1.85, p=0.17, partial η2=0.02).

**Supplementary tables and figures**

**Supplementary Table S1** Neuropsychological tests in SA and TA groups.

| **Test** | **TA (n=110)** | | **SA-ALL (n=74)** | | **SA-CAG (n=37)** | | **SA-EM (n=37)** | | **SA-NM (n=37)** | | **SA-CVLT (n=31)** | |
| --- | --- | --- | --- | --- | --- | --- | --- | --- | --- | --- | --- | --- |
|  | **Mean (SD)** | **Median (IQR)** | **Mean (SD)** | **Median (IQR)** | **Mean (SD)** | **Median (IQR)** | **Mean (SD)** | **Median (IQR)** | **Mean (SD)** | **Median (IQR)** | **Mean (SD)** | **Median (IQR)** |
| CVLT Trials 1-5 FR | 42.54 (7.84) | 42.00 (11.00) | 51.85 (10.35) | 52.00 (14.00) | 52.46 (10.78) | 52.00 (14.00) | 53.05 (10.55) | 52 (16.00) | 52.11 (11.12) | 52.00 (16) | 59.03 (7.61) | 58.00 (10.00) |
| CVLT SDFR | 8.33 (2.87) | 9.00 (3.00) | 11.36 (3.25) | 12 (5.00) | 11.16 (3.39) | 11.00 (5.00) | 11.41 (3.30) | 12.00 (5.00) | 11.49 (3.51) | 12.00 (5.00) | 13.87 (1.80) | 14.00 (2.00) |
| CVLT SDCR | 10.11 (2.84) | 11.00 (3.00) | 12.73 (2.51) | 13.50 (4.00) | 12.54 (2.71) | 13.00 (4.00) | 12.78 (2.66) | 14.00 (4.00) | 12.68 (2.75) | 13.00 (5.00) | 14.65 (1.14) | 15.00 (2.00) |
| CVLT LDFR | 8.99 (2.66) | 10.00 (4.00) | 12.23 (2.98) | 13.00 (3.00) | 11.76 (3.18) | 12.00 (4.00) | 12.32 (2.87) | 13.00 (4.00) | 12.05 (3.11) | 13.00 (5.00) | 14.81 (0.83) | 15.00 (2.00) |
| CVLT LDCR | 10.11 (2.76) | 10.00 (3.00) | 12.78 (2.53) | 13.00 (4.00) | 12.54 (2.73) | 13.00 (4.00) | 12.92 (2.53) | 13.00 (4.00) | 12.43 (2.97) | 13.00 (4.00) | 14.84 (0.90) | 15.00 (1.00) |
| Logical Memory | 41.12 (8.45) | 41.00 (12.00) | 47.22 (7.74) | 47.22 (48.00) | 47.51 (8.49) | 49.00 (10.00) | 49.11 (7.56) | 50.00 (10.00) | 47.35 (6.29) | 48.00 (8.00) | 46.68 (7.46) | 49.00 (10.00) |
| VR I | 66.01 (13.98) | 67.00 (19.00) | 80.34 (12.57) | 84.00 (19.00) | 82.08 (11.20) | 85.00 (20.00) | 85.97 (9.26) | 89.00 (11.00) | 80.41 (11.76) | 82.00 (18.00) | 79.19 (13.47) | 82.00 (18.00) |
| VR II | 43.75 (18.16) | 43.00 (26.00) | 63.66 (17.43) | 66.50 (26.00) | 64.08 (16.88) | 67.00 (26.00) | 73.84 (12.79) | 74.00 (17.00) | 61.84 (15.54) | 64.00 (25.00) | 63.9 (16.38) | 65.00 (22.00) |
| VR Recognition | 41.40 (4.07) | 42.00 (6.00) | 44.61 (2.32) | 45.00 (3.00) | 44.08 (2.44) | 45.00 (3.00) | 45.43 (1.80) | 45.00 (2.00) | 44.35 (2.23) | 45.00 (3.00) | 44.58 (2.38) | 45.00 (4.00) |
| TMT-A | 43.27 (16.28) | 39.91 (17.22) | 35.67 (9.88) | 33.61 (11.54) | 35.83 (7.68) | 35.66 (9.88) | 36.32 (8.71) | 33.62 (11.78) | 32.90 (8.19) | 32.25 (10.85) | 36.16 (10.32) | 34.71 (11.35) |
| TMT-B | 96.69 (45.51) | 83.45 (34.86) | 69.48 (21.11) | 67.10 (26.24) | 67.92 (20.17) | 66.28 (27.67) | 72.97 (18.43) | 72.66 (24.14) | 57.41 (12.65) | 57.62 (19.21) | 67.27 (21.66) | 66.78 (26.28) |
| Stroop in 60 s | 43.65 (11.07) | 44.00 (15.00) | 50.74 (10.82) | 50.50 (15.00) | 51.65 (10.47) | 51.00 (15.00) | 49.92 (10.60) | 49.00 (14.00) | 55.35 (9.22) | 56.00 (13.00) | 52.10 (10.97) | 50.00 (14.00) |
| FAS test | 43.10 (11.67) | 43.00 (15.00) | 48.84 (10.58) | 48.00 (17.00) | 49.65 (10.93) | 49.00 (18.00) | 47.14 (9.92) | 46.00 (13.00) | 53.59 (7.84) | 53.00 (11.00) | 51.45 (11.08) | 50.00 (17.00) |
| Animal Naming | 18.59 (4.96) | 19.00 (7.00) | 20.66 (4.07) | 20.00 (5.00) | 20.95 (4.40) | 20.00 (6.00) | 20.59 (4.17) | 20.00 (6.00) | 21.35 (4.11) | 21.00 (5.00) | 21.77 (4.37) | 21.00 (7.00) |
| Vegetable Naming | 12.76 (3.65) | 13.00 (4.00) | 15.72 (3.88) | 16.00 (5.00) | 15.84 (3.79) | 16.00 (6.00) | 15.14 (4.28) | 15.00 (5.00) | 16.73 (4.13) | 16.00 (7.00) | 17.16 (3.12) | 17.00 (6.00) |
| Digit Symbol | 51.15 (9.65) | 51.50 (13.00) | 64.07 (9.46) | 65.00 (13.00) | 67.76 (9.10) | 67.00 (11.00) | 62.65 (9.11) | 63.00 (11.00) | 68.35 (8.85) | 67.00 (11.00) | 64.06 (10.81) | 65.00 (16.00) |
| DSF | 8.26 (2.22) | 8.00 (3.00) | 8.74 (2.45) | 9.00 (4.00) | 9.43 (2.36) | 9.00 (4.00) | 9.14 (2.45) | 9.00 (4.00) | 9.32 (2.59) | 9.00 (4.00) | 8.71 (2.34) | 9.00 (4.00) |
| DSB | 6.98 (1.65) | 7.00 (2.00) | 7.70 (2.75) | 7.00 (4.00) | 7.38 (2.61) | 7.00 (4.00) | 7.78 (2.51) | 8.00 (4.00) | 8.35 (2.61) | 8.00 (4.00) | 7.81 (2.77) | 8.00 (5.00) |
| BNT | 14.46 (0.81) | 15.00 (1.00) | 14.66 (0.58) | 15.00 (1.00) | 14.73 (0.51) | 15.00 (1.00) | 14.73 (0.51) | 15.00 (1.00) | 14.84 (0.44) | 15.00 (0.00) | 14.65 (0.61) | 15.00 (1.00) |

BNT: Boston Naming Task; CAG: cognitive age gap; CVLT: California Verbal Learning Test; DSB: digit span backward; DSF: digit span forward; EM: episodic memory; FR: free recall total; IQR: interquartile range; LDCR: Long-Delay Cued Recall; LDFR: Long Delay Free Recall; NM: non-memory cognition; SDCR: Short-Delay Cued Recall; SDFR: Short Delay Free Recall; SA: successful agers; TA: typical agers; TMT: Trail Making Test; VR: Visual Reproduction.

**
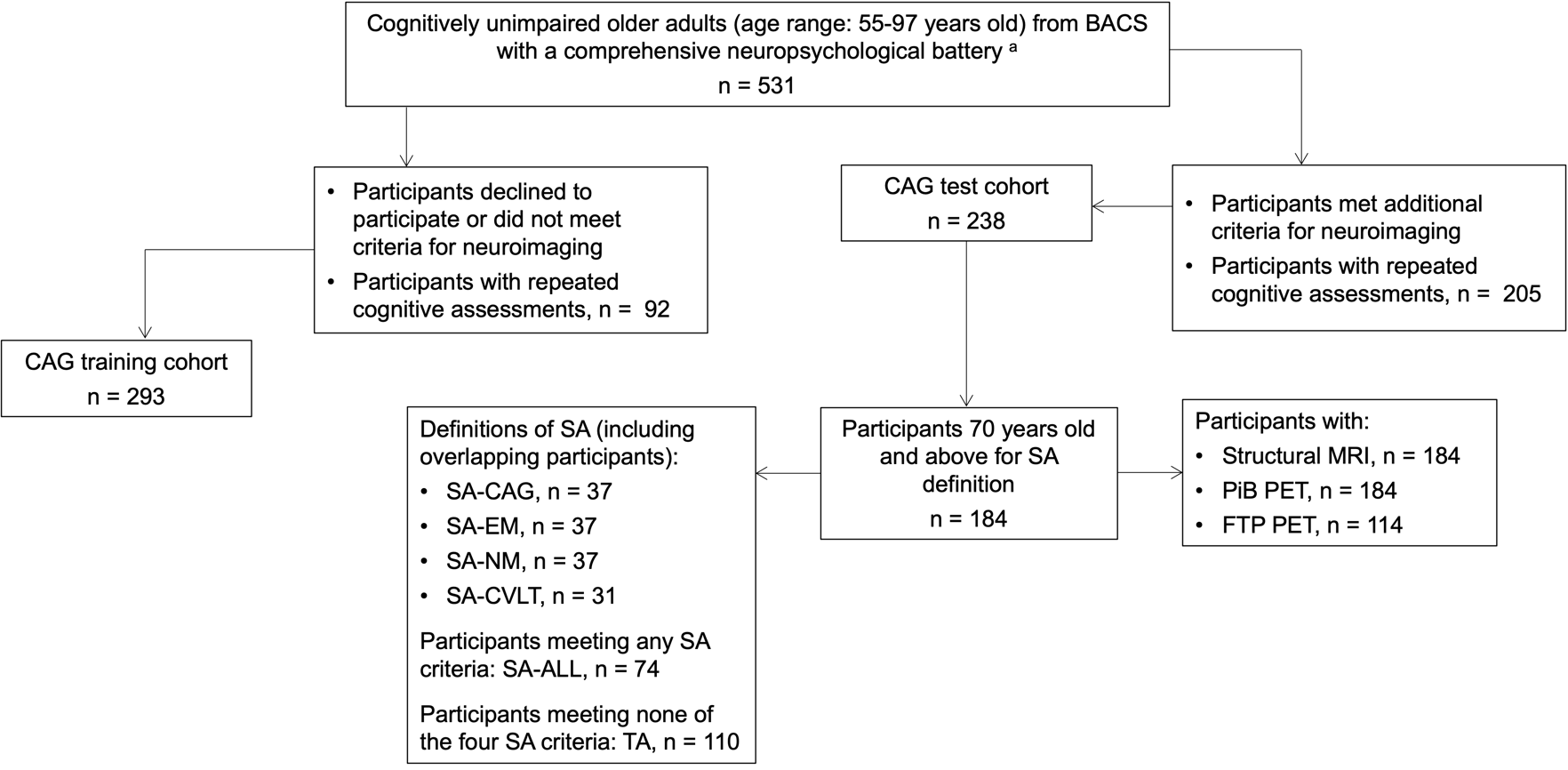
**

**Supplementary Figure S1** Flow chart describing the selection process of BACS participants. ^a^ Neuropsychological tests: CVLT, Logical Memory, Visual Reproduction, TMT-A and B, Stroop test, digit symbol task, phonemic verbal fluency FAS test, Animal Naming, Vegetable Naming, digit span forward and backward. BACS: Berkeley Aging Cohort Study; CAG: cognitive age gap; CVLT: California Verbal Learning Test; EM: episodic memory; FTP: ^18^F-Flortaucipir; MMSE: Mini-Mental State Examination; MRI: structural magnetic resonance imaging; NM: non-memory cognition; PET: positron emission tomography; PiB: ^11^C-Pittsburgh compound B; SA: successful agers; TA: typical agers; TMT: Trail Making Test.

**
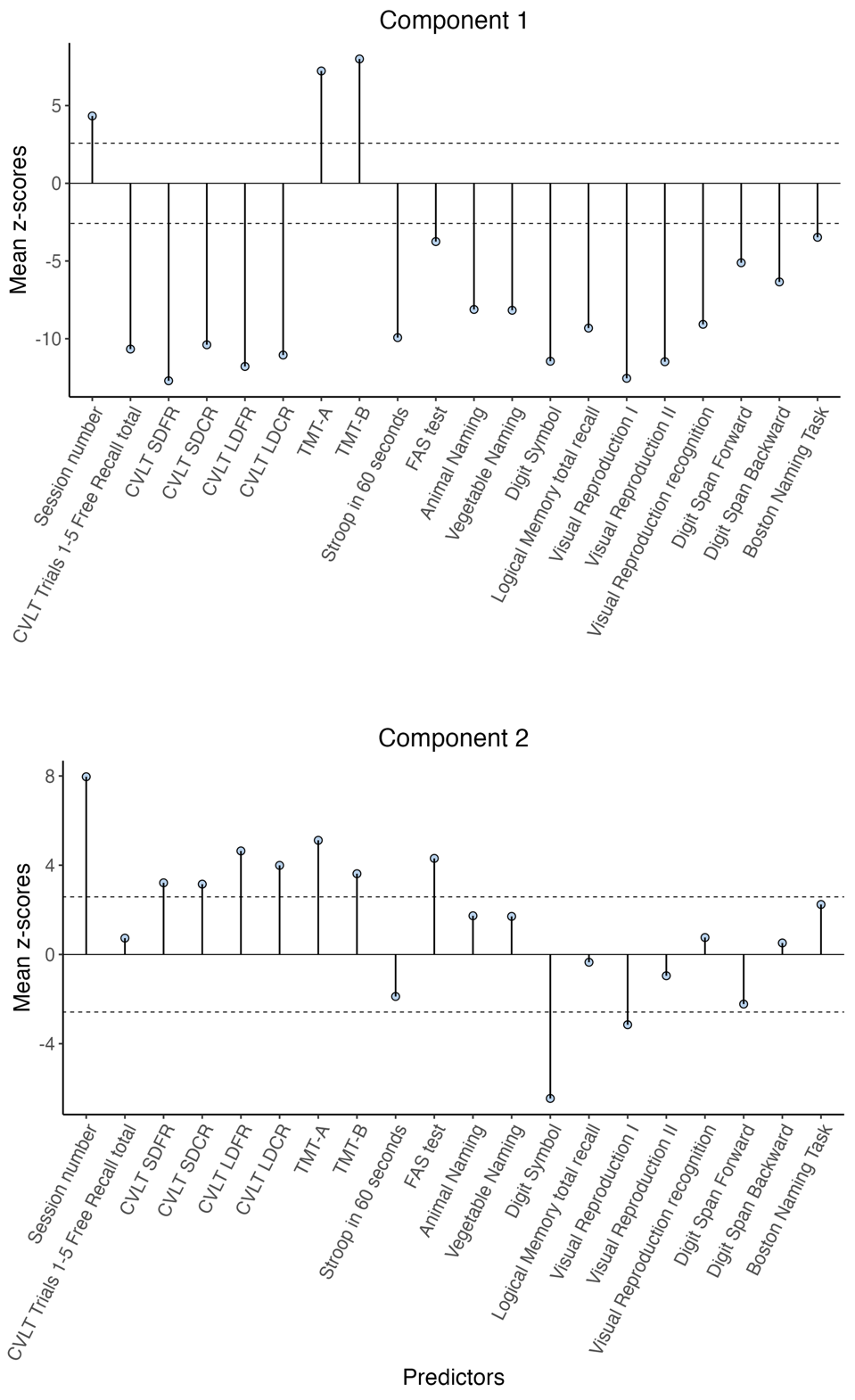
**

**Supplementary Figure S2** Normalized weights for component 1 and 2 showing that all predictors significantly contributed to component 1, and 10 out of 19 predictors significantly contributed to component 2. Dashed lines represent the significance threshold set at p<0.01 corresponding to z-scores < -2.58 or > +2.58. CVLT: California Verbal Learning Test; LDCR: Long-Delay Cued Recall; LDFR: Long Delay Free Recall; SDCR: Short-Delay Cued Recall; SDFR: Short Delay Free Recall; TMT: Trail Making Test.

**
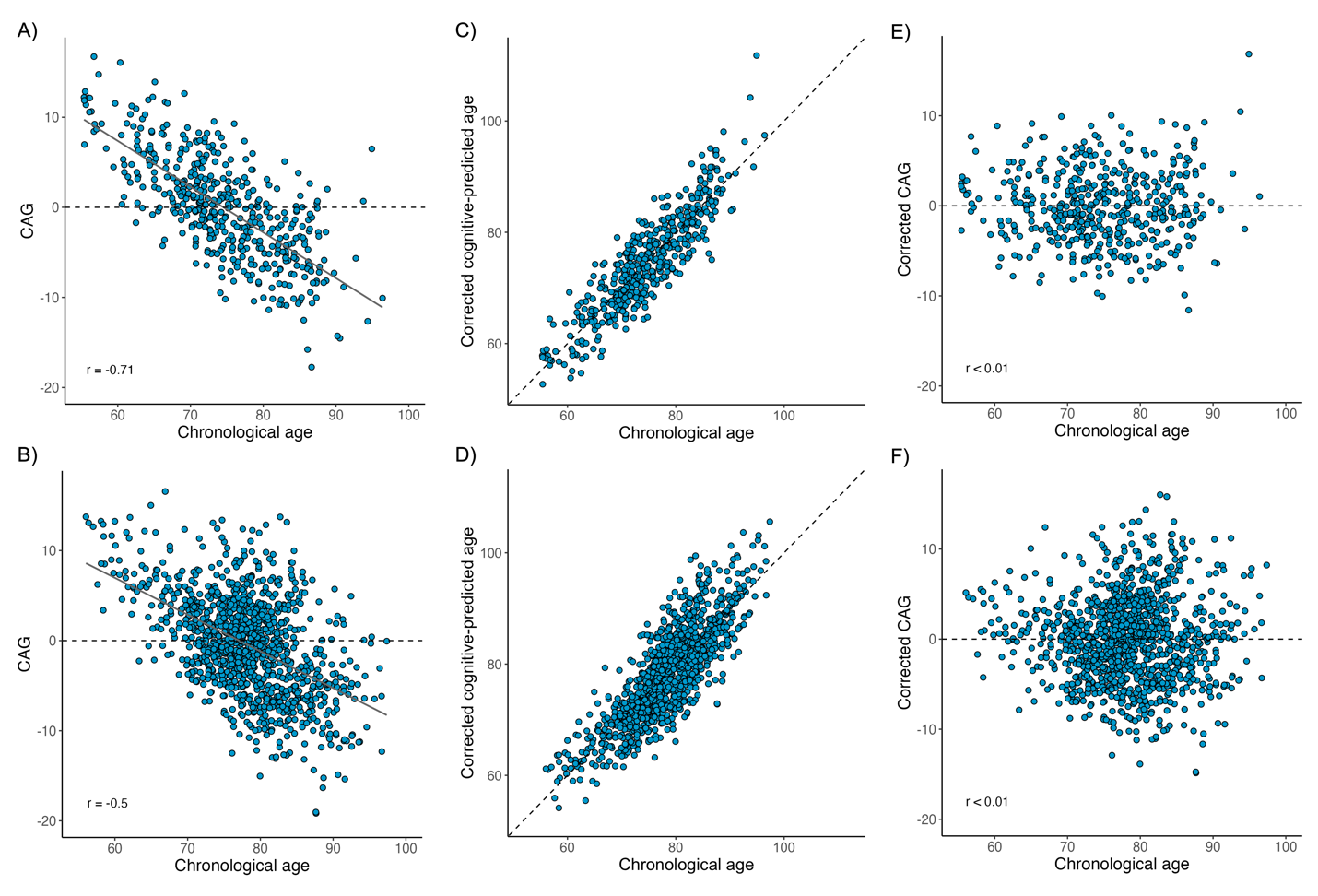
**

**Supplementary Figure S3** Age-bias correction applied to the training (top row) and testing (bottom row) cohorts. Scatterplots showing: A), B) Association between CAG estimates and chronological age in training (A) and test (B) cohorts (the gray lines are the regression lines of chronological age on CAG) showing age-related bias. C), D) Association between cognitive-predicted and chronological age after age-bias correction (coefficients from the linear fit shown in A and B were used to correct age predictions, see Methods for details) in training (C) and test (D) samples (the dashed lines represent the line of identity (x=y) where cognitive-predicted age = chronological age). E), F) Relationships between corrected CAG (calculated as corrected cognitive-predicted age – chronological age) showing no significant association in training (E) and test (F) cohorts.
